# A Case of Two Brothers With Familial Frontotemporal Dementia Caused by a Progranulin Gene Mutation: A Case Report

**DOI:** 10.1192/bjo.2026.11897

**Published:** 2026-06-30

**Authors:** Alexander Sunderland, Elizabeth Emmett, Nancy Youssef, Tarun Kuruvilla

**Affiliations:** Gloucestershire Health and Care NHS Foundation Trust, Gloucester, United Kingdom

## Abstract

**Aims:**

Frontotemporal dementia (FTD) is the second most common cause of early-onset dementia accounting for approximately 10–20% of all dementia cases. The behavioural variant (bvFTD) is the most common presentation, comprising 50–57% of autopsy-confirmed FTD cases. bvFTD is highly heritable, and most implicated genes are MAPT, GRN (progranulin), and C9orf72.

**Methods:**

We report a case of two brothers, the first brother, aged 62, was assessed in the memory service in 2022 following a five-year history of behavioural change including inappropriate behaviour, overeating, apathy, and memory impairment. His Addenbrooke’s Cognitive Examination (ACEIII) score was 56/100. CT brain imaging showed generalised atrophy, more pronounced on the right. PET CT demonstrated hypometabolism particularly in the right frontotemporal region. Amyloid PET imaging showed no abnormal amyloid binding.

The second brother, aged 64, was assessed in 2024 with a two-year history of progressive cognitive decline and pronounced frontal lobesymptoms including apathy, irritability, lack of insight, impaired executive function, and poor financial management leading to debt. He required family support for most activities of daily living and scored 24/100 on the ACE. CT brain imaging showed pronounced frontal and medial temporal lobe atrophy. FDG-PET demonstrated reduced uptake in the frontal, temporal, and parietal lobes.

Both brothers underwent whole genome sequencing and were found to be heterozygous for a GRN mutation. Predictive genetic testing was recommended to their children, and two of four tested positive for the mutation.

**Results:**

GRN mutations account for 35% of genetic FTD and around 10% of all FTD cases. GRN-FTD follows an autosomal dominant inheritance pattern. In our patients, there was no family history of FTD, although their father had possible undiagnosed dementia while their mother had Parkinson’s disease and Alzheimer’s disease. Notably, up to 13% of bvFTD cases carry a genetic variant despite no family history.

Genetic testing is a crucial diagnostic tool for defining the molecular pathology of the disease particularly in psychiatric settings where early bvFTD is often misdiagnosed as a primary psychiatric disorder. Furthermore, Identification of a GRN mutation is also essential for determining eligibility for clinical trials.

Predictive genetic testing for at-risk relatives enables them to make informed decisions regarding life and future care.

**Conclusion:**

This case highlights the importance of whole genome sequencing and predictive genetic testing even in the absence of a convincing family history. This could improve both patient care and our overall understanding of the disease.

